# Prevalence of substance use disorder in individuals with attention deficit/hyperactivity disorder: associations with sex and psychiatric comorbidity

**DOI:** 10.1186/s12888-025-07305-1

**Published:** 2025-10-07

**Authors:** Camilla Djønne Moldekleiv, Astri J. Lundervold, Berit Skretting Solberg, Anders Engeland, Lars Thore Fadnes, Fatemeh Chalabianloo, Kari Klungsøyr

**Affiliations:** 1 Department of Biological and Medical Psychology, University of Bergen, Bergen, Norway; 2 Department of Biomedicine, University of Bergen, Bergen, Norway; 3 Child- and Adolescent Psychiatric Outpatient Unit, Hospital Betanien, Bergen, Norway; 4 Department of Global Public Health and Primary Care, University of Bergen, Postbox 7800, Bergen, 5020 Norway; 5 Division of Public Health and Prevention, Norwegian Institute of Public Health, Zander Kaaes Gate 7, Bergen, 5015 Norway; 6 Department of Addiction Medicine, Bergen Addiction Research, Haukeland University Hospital, Bergen, Norway

**Keywords:** Attention-deficit/hyperactivity disorder, Substance use disorder, ADHD, SUD, Sex, Gender, Psychiatric disorders, Comorbidity

## Abstract

**Background:**

Several studies have reported associations between attention-deficit/hyperactivity disorder (ADHD) and substance use disorders (SUD). However, sex-differences in ADHD and SUD, especially when associated with other psychiatric comorbidities, are less studied.

**Objective:**

To compare the prevalence of SUD among individuals with and without ADHD to describe groups, defined by sex and psychiatric comorbidity, who may benefit from targeted follow-up.

**Methods:**

Data from the Medical Birth Registry of Norway, the Norwegian Prescription Database and the Norwegian Patient Registry were linked. Individuals born 1988–2001 were identified and followed until 2019 (ages 18–31 years in 2019). Log binomial regression was used to calculate age-adjusted prevalence differences (PD) of SUD with 95% confidence intervals in males (*N* = 31,146) and females (*N* = 18,669) with ADHD, overall and with/without other psychiatric comorbidities, relative to those without ADHD (males *N* = 381,568; females *N* = 368,804).

**Results:**

ADHD was associated with SUD across all substances among both males and females (“Any SUD”: PD_females_ 10.9% (10.4; 11.4); PD_males_ 11.4% (11.0; 11.8). Compared to individuals without ADHD, PDs were larger in males with ADHD than females for disorders related to cannabis, sedatives, stimulants, other substances and multiple psychoactive substances. ADHD with psychiatric comorbidities was associated with even larger differences in SUD prevalence among both males and females, the largest for ADHD with psychosis/schizophrenia [PD_males_ 49.0% (45.6; 52.4); PD_females_ 40.6% (36.1; 45.1)] and ADHD with personality disorders [PD_males_ 37.6% (35.2; 40.0); PD_females_ 33.0% (31.0; 35.0)], compared to males and females without psychiatric disorders. For psychiatric comorbidities typically diagnosed in adulthood, PDs were larger in males than females with ADHD, whereas for comorbid childhood disorders, PDs were larger in females.

**Conclusion:**

The high prevalence of SUD associated with ADHD, with higher PDs in males than females for most specific SUDs, indicates a need for targeted prevention. The substantially higher prevalence of SUD in individuals with ADHD and comorbid psychiatric disorders emphasizes the need for close follow-up of individuals with ADHD both regarding SUD and other psychiatric disorders. Further studies are needed to evaluate potential causal relations between ADHD, other psychiatric comorbidities, and SUD, and to understand the sex-differences in SUD prevalence across psychiatric comorbidities in ADHD.

**Supplementary Information:**

The online version contains supplementary material available at 10.1186/s12888-025-07305-1.

## Background

Substance use disorder (SUD) is an umbrella term used to describe dependency and harmful use of alcohol and substances such as opioids, cannabinoids, sedatives and stimulants [1, 2]. Globally, the prevalence of SUD is increasing, and it is considered a major public health problem [3, 4]. In Norway, around 8% of adult males and 3% of adult females are found to have a substance use related problem [2]. SUD often occurs together with other psychiatric disorders, with attention-deficit/hyperactivity disorder (ADHD), anxiety-, mood-, conduct- and personality disorders described as the most common comorbidities [5–7].

ADHD is a highly heritable neurodevelopmental disorder [8]. Although it has a childhood onset, impairing symptoms often persist into adulthood [8–10] with prevalence rates around 6% in childhood/adolescence and 3% in adulthood [11]. ADHD is defined by symptoms of inattention and/or hyperactivity-impulsivity that interferes with normal development [12], and individuals with ADHD commonly experience challenges leading to psychological distress, reduced quality of life, and educational and occupational underachievement [13–15]. The male to female prevalence ratio of ADHD changes with age; from 2–3:1 in childhood to 1.3:1 in adults (population-based samples) [16–18]. This has been suggested to reflect that ADHD to a lesser degree is detected in girls at an early age [18–20], and that the diagnostic criteria may better fit the symptoms shown by young males than young females, who often have a slightly different presentation with less hyperactivity and impulsivity [18, 21].

A meta-analysis from 2012 showed that 25% of adolescents and 21% of adults with SUD had co-occurrent ADHD [22]. This comorbidity is linked with lower remission rates of SUD, more severe and longer duration of SUD, and less successful recovery with SUD treatment [22–26]. Another meta-analysis, including 27 studies from 1990–2008, showed that children with ADHD had a significantly increased risk of developing SUD during their lifetime compared to children without ADHD [27]. ADHD also tends to appear together with other psychiatric disorders [12]. Anxiety disorders, conduct disorders and oppositional disorders are commonly described in childhood cases [28–30] and mood, anxiety and personality disorders are common in adults and adolescents [17, 31, 32]. As mentioned above, SUD frequently co-occurs with ADHD [22, 23, 27, 33].

Most studies on co-occurring ADHD and SUD have included small samples, and only a few studies have included females [34–39]. Some suggested that females with ADHD have higher risk of SUD than males with ADHD [35] and higher risk of more severe psychiatric disorders, including schizophrenia [35, 38]. However, these previous studies included small sample sizes, they reported only relative effect measures, and few studies reported sex-specific results. Four large population-based studies have reported sex-specific associations between ADHD and SUD [17, 34, 40, 41], where two reported both relative and absolute effect measures [17, 41]. Using relative effect measures, Ottosen et al. [40] and Chen et al. [41] both reported higher increase in risk of SUD in females than males with ADHD, while Solberg et al. [17] found no clear sex-difference. In an earlier study by Ottosen et al. [34] females with ADHD were found to have higher increase in risk of alcohol and cannabis abuse than males, but not when adjusting for additional psychiatric comorbidities. On the absolute scale, Solberg et al. [17] and Chen et al. [41] reported larger differences of SUD prevalence in males with versus without ADHD than females, in contrast to other psychiatric comorbidities where difference in prevalence was larger in females with versus without ADHD than males.

While relative effect measures (risk ratios) are helpful when investigating causal relations, absolute effect measures (differences in risk), are better when the aim is to report results with clinical and public health relevance, e.g. to identify groups of individuals that could profit most from preventive measures [42, 43].

The present study will therefore use absolute effect measures (risk differences) to compare the prevalence of SUD among individuals with and without ADHD with an overarching goal of describing groups of individuals, defined by sex and psychiatric comorbidity, who may benefit from targeted prevention and closer follow up.

## Methods

The present study is a national registry-based study using linked data from the Medical Birth Registry of Norway (MBRN), the Norwegian Prescription Database (NorPD), the Norwegian Patient Registry (NPR) and the National Education Database, all based on mandatory reporting. The MBRN, established in 1967, registers all births in Norway from 12–16 gestational weeks, with information on the mother, father, pregnancy, delivery and the child [44]. It is routinely linked with the National Population Registry through which all dates of death and emigration are included. The NorPD, established in 2004, includes information on all prescribed and dispensed medications from any pharmacy to individuals in Norway, using the Anatomical Therapeutic Chemical (ATC) classification system [45]. The registry includes information on reimbursement, and from 2008, also detailed information on the indication for reimbursed medication. The NPR, established in 1997, includes administrative, demographic, and medical information reported from specialist health care [46]. The International Statistical Classification of Diseases, 10th Revision (ICD-10) is used for coding diagnostic data [47]. From 2008, the national identification numbers have been included in the registry, enabling linkage between NPR and other registries. The NED, established in 1970, contains information on educational attainment in Norway for citizens aged 16 years or more [48].

From the MBRN, we included all individuals born alive between 1988 and 2001 and followed them to 2019 with data from the NorPD and the NPR, by using the national identification number unique to every Norwegian resident for linkage. To analyze sex-differences, we only had data on sex from the MBRN, assigned at birth. We have therefore used the term “sex” and not “gender” throughout the text.

We defined individuals with ADHD as those who had been dispensed ADHD-medication during 2004–2019 (NorPD) or had an ADHD-diagnosis (ICD-10: F90) registered in the NPR during 2008–2019. The ADHD-medications identified by their ATC codes were the central stimulants methylphenidate (N06BA04), amphetamine (N06BA01), dexamphetamine (N06BA02), and lisdexamphetamine (N06BA12), and the non-stimulant drug atomoxetine (N06BA09). Individuals who had been prescribed central stimulants for narcolepsy from 2008 were excluded. Individuals who died or emigrated before 2008 when the NPR was made personally identifiable were also excluded as were those who died before the age of 10 years, since a SUD diagnoses before this age is assumed to be extremely rare.

The SUD-diagnoses were based on data from the NPR, using ICD-10 codes from the F1-chapter. The following SUD-diagnoses were studied: alcohol-related disorders (F10), opioid-related disorders (F11), cannabis-related disorders (F12), sedative-related disorders, e.g. benzodiazepines (F13), stimulant-related disorders, e.g. cocaine and amphetamines (F14-F15), other substance related disorders: hallucinogens (F16) and volatile solvents (F18), and multiple drug use/other psychoactive substances (F19) [47]. Nicotine dependency (F17) was not included, as we assume it is rarely used as a clinical diagnosis and therefore highly underreported in the registers. Other psychiatric disorders were also based on ICD-10 codes registered in the NPR, and the following were studied: schizophrenia/psychosis (F2), mood/affective disorders (F3), anxiety/somatoform disorders (F4), eating/sleeping disorders (F5), personality disorders (F6), disorders of psychological development, including learning disabilities and autism spectrum disorders (F8), conduct disorders (F91), mixed disorders of conduct and emotion (F92), and emotional disorders with childhood onset (F93) [47].

### Statistical analyses

We used log binomial regression and calculated absolute prevalence differences (PD) of SUD with 95% confidence intervals (CI) in individuals with ADHD versus individuals without (non-ADHD). To take account of correlations between siblings in our study population, we used clustered robust standard errors with mother’s ID as cluster. All analyses were adjusted for birth year (by 2-year categories). Our main aim was to describe which groups (by sex and psychiatric comorbidity) had the highest SUD prevalence, and not to identify underlying causal mechanisms. Thus, we did not adjust for other covariates when analyzing PDs. With the same reasoning, the most important measures in our study were absolute prevalence rates and PDs [42, 43].

However, we also applied Cox proportional hazards regression models for hazard ratios (HR) with 95% CI to enable comparison with previous studies. In these analyses, individuals were followed from 2008 (when NPR was made identifiable) to the first registered SUD diagnosis, their death or the end of study in 2019, whatever came first. In these models, we adjusted for birth year, mother’s educational level at the individual’s birth (low, middle, high) and, when analyzing the total population, also for sex. We used clustered robust standard errors with mother’s ID as cluster to take account of correlations between siblings.

Interactions by sex were tested on both the additive and multiplicative scales [43].

Analyses were carried out using STATA (i.c.18). The study was approved by the Regional Ethics Committee in Norway (2020/75421). For analysis of de-identified registry data, no consent was required.

## Results

We identified a total of 800,187 individuals who were born during 1988–2001 and were alive and living in Norway in 2008 (females: *N* = 387,473; males: *N* = 412,714). Approximately 48% of the individuals in this study population did not have siblings, 39% had one sibling and 13% had two or more siblings. Overall, 49,815 (6.2%) of individuals were registered with ADHD (males: *N* = 31,146; 7.5%; females: *N* = 18,669; 4.8%; Table 1). This population contributed more than 8 million person-years of follow-up for the SUD outcomes.

**Table 1:** Characteristics of males and females with and without ADHD and their mothers

Characteristics	Individuals without ADHD			Individuals with ADHD		
	Total: N (%)	Females: N (%)	Males: N (%)	Total: N (%)	Females: N (%)	Males: N (%)
Total	750,372	368,804	381,568	49,815 (6.2)	18,669 (4.8)	31,146 (7.5)
Individual’s years of age in 2019 (mean, SEM)	24.5 (0.005)	24.5 (0.007)	24.5 (0.006)	24.3 (0.02)	24.6 (0.02)	24.1 (0.02)
Born preterm (< 37 gestational weeks)	41,827 (6.0)	19,327 (5.7)	22,500 (6.4)	3,504 (7.7)	1,146 (6.8)	2,358 (8.3)
Twin/triplet	21,774 (2.9)	10,852 (2.9)	10,922 (2.9)	1,345 (2.7)	460 (2.5)	885 (2.8)
Individuals with other psychiatric disorders						
*Schizophrenia, psychosis (F2)*	5,048 (0.7)	2,358 (0.6)	2,923 (0.8)	1,290 (2.6)	465 (2.5)	825 (2.7)
*Mood/affective disorders (F3)*	64,643 (8.6)	43,184 (11.7)	21,459 (5.6)	12,060 (24.2)	6,663 (35.7)	5,397 (17.3)
*Anxiety/somatoform disorders (F4)*	90,701 (12.1)	60,845 (16.5)	29,856 (7.8)	15,979 (32.1)	8,585 (46.0)	7,394 (23.7)
*Eating/sleeping disorders (F5)*	15,537 (2.1)	13,023 (3.5)	2,514 (0.7)	2,410 (4.8)	1,692 (9.1)	718 (2.3)
*Personality disorders (F6)*	14,231 (1.9)	8,824 (2.4)	5,407 (1.4)	3,878 (7.8)	2,263 (12.1)	1,615 (5.2)
*Disorders of psychological development (F8)*	17,876 (2.4)	6,927 (1.9)	10,949 (2.9)	12,606 (25.3)	4,158 (22.3)	8,448 (27.1)
*Conduct disorder (F91)*	2,254 (0.3)	746 (0.2)	1,508 (0.4)	1,656 (3.3)	476 (2.6)	1,180 (3.8)
*Mixed disorder of conduct and emotion (F92)*	2,188 (0.3)	1,006 (0.3)	1,182 (0.3)	1,044 (2.1)	389 (2.1)	655 (2.1)
*Emotional disorder with onset specific to childhood (F93)*	5,579 (0.7)	3,166 (0.9)	2,413 (0.6)	1,649 (3.3)	710 (3.8)	939 (3.0)
Maternal age (years) when giving birth to the individual						
< 20	23,999 (3.2)	12,009 (3.3)	11,990 (3.1)	3,457 (6.9)	1,232 (6.6)	2,225 (7.1)
20–34	638,378 (85.1)	313,672 (85.1)	324,706 (85.1)	41,754 (83.8)	15,684 (84.0)	26,070 (83.7)
35 +	87,995 (11.7)	43,123 (11.7)	44,872 (11.8)	4,604 (9.2)	1,753 (9.4)	2,851 (9.2)
Maternal parity: Nulliparous	308,141 (41.1)	151,751 (41.2)	156,390 (41.0)	21,774 (43.7)	8,010 (42.9)	13,764 (44.2)
Maternal cohabitation: Single	56,814 (7.6)	28,293 (7.7)	28,521 (7.5)	7,954 (16.1)	2,987 (16.2)	4,967 (16.1)
Maternal education: Low	142,048 (19.1)	70,345 (19.2)	71,703 (18.9)	14,061 (28.4)	5,298 (28.5)	8,763 (28.3)
Middle	305,325 (41.0)	150,239 (41.0)	155,086 (40.9)	20,510 (41.4)	7,595 (40.9)	12,915 (41.7)
High	298,301 (40.0)	145,955 (39.8)	152,346 (40.2)	14,946 (30.2)	5,680 (30.6)	9,266 (29.9)

The percentage of individuals born preterm was higher among those with than without ADHD (7.7% versus 6.0%) and a higher percentage of individuals with ADHD were born to mothers who were single (16.1% versus 7.6%), had low educational level (28.4% versus 19.1%) and were less than 20 years when giving birth to the individual (6.9% versus 3.2%, Table 1). The prevalence rates of other comorbid psychiatric disorders were higher among individuals with than without ADHD, with anxiety/somatoform disorders as the most common, followed by disorders of psychological development. There were further evident sex-differences in the prevalence of psychiatric disorders both among individuals with and without ADHD. Females had higher prevalence than males of mood/affective disorders, anxiety/somatoform disorders, eating/sleeping disorders, personality disorders and emotional disorders with childhood onset, while males had higher prevalence of schizophrenia/psychosis, disorders of psychological development, and conduct disorders.

### Prevalence of SUD in individuals with versus without ADHD

**Fig. 1:**
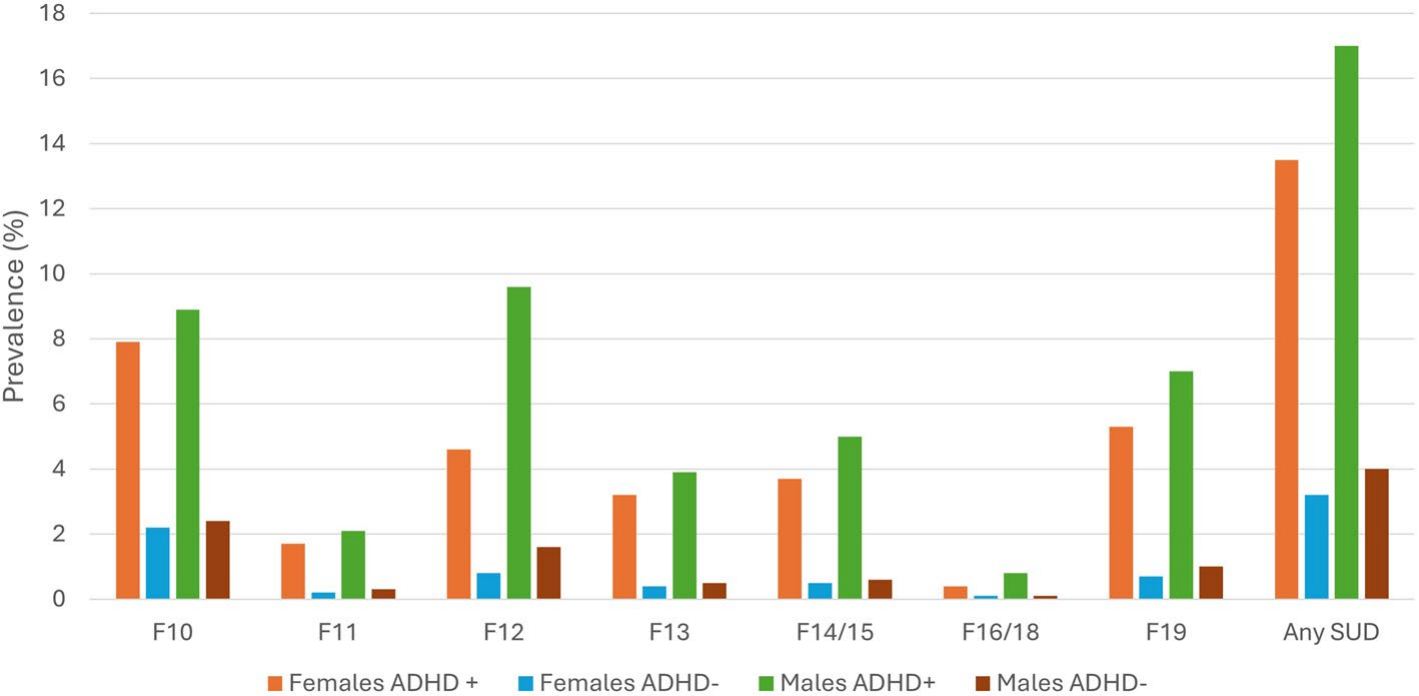
Age adjusted prevalence of substance use disorders in males and females with and without ADHD

We identified 34,883 (4.4%) individuals registered with any SUD in specialist care (males: 20,571 (5.0%); females: *N* = 14,312 (3.7%)). The age-adjusted prevalence rates of various SUD diagnoses in males and females with and without ADHD are shown in Fig. 1 and Additional material, Additional file 1. Higher age-adjusted rates were found across all SUD diagnoses in both males and females with ADHD compared to males and females without. Further, independent of ADHD, there were consistently higher age-adjusted prevalence rates in males than females. The highest age-adjusted rates were found for cannabis-related disorders in males with ADHD (9.58%; 95% CI: 9.27–9.88) and for the group “any SUD” in males with ADHD (17.01%; 16.65–17.37); see Additional file 1.

Overall, individuals with ADHD were more frequently given SUD diagnoses in specialist care than individuals without ADHD (Fig. 1 and Additional material, additional file 2). Sex-specific PD estimates, presented in Table 2, showed that the largest differences in age-adjusted prevalence in both males and females with versus without ADHD were for"any SUD": PD_females_ 10.9%; PD_males,_ 11.4%. The smallest differences were found for other substance related disorders including hallucinogens and volatile solvents and opioid-related disorders. There were significantly larger ADHD-associated PDs among males than females for disorders related to cannabis, sedatives, stimulants (cocaine and amphetamines), other substances including hallucinogens and volatile solvents and multiple drug use/other psychoactive substances.

**Table 2:** Prevalence differences and hazard ratios of SUD in males and females with and without ADHD

Substance	Females				Males			
	Non-ADHD N (%)	ADHD N (%)	PD^a^ (95% CI)	HR^b^ (95% CI)	Non-ADHD N (%)	ADHD N (%)	PD^a^ (95% CI)	HR^b^ (95% CI)
Alcohol-related disorders (F10)	7,870 (2.1)	1,560 (8.4)	6.1 (5.7; 6.4)	3.8 (3.6; 4.0)	9,326 (2.4)	2,620 (8.4)	5.7 (5.4; 6.0)	3.5 (3.3; 3.6)
Opioid- related disorders (F11)	771 (0.2)	316 (1.7)	1.4 (1.2; 1.6)	7.4 (6.4; 8.5)	1,018 (0.3)	609 (2.0)	1.5 (1.4; 1.7)	7.6 (6.8; 8.4)
Cannabis- related disorders (F12)	2,736 (0.7)	971 (5.2)	4.3 (4.0; 4.6)^c^	6.5 (6.0; 7.1)^d^	6,325 (1.7)	2,740 (8.8)	6.8 (6.5; 7.1)^c^	5.5 (5.2; 5.7)^d^
Sedatives- related disorders (e.g., benzodiazepines) (F13)	1,561 (0.4)	566 (3.0)	2.5 (2.3; 2.7)^c^	6.8 (6.1; 7.5)	1,951 (0.5)	1,165 (3. 7)	3.0 (2.8; 3.2)^c^	7.5 (6.9; 8.1)
Stimulant- related disorders (e.g., cocaine and amphetamines) (F14-F15)	1,678 (0.5)	733 (3.9)	3.4 (3.1; 3.6)^c^	8.0 (7.3; 8.8)	2,475 (0.7)	1,422 (4.6)	3.7 (3.5; 3.9)^c^	7.3 (6.8; 7.8)
Other substance- related disorders (F16, F18)	207 (0.1)	76 (0.4)	0.3 (0.3; 0.4)^c^	6.7 (5.1; 8.8)	468 (0.1)	228 (0.7)	0.6 (0.5; 0.7)^c^	5.7 (4.9; 6.8)
Multiple psychoactive substance-related disorders (F19)	2,702 (0.7)	1,019 (5.5)	4.6 (4.3; 4.9)^c^	6.8 (6.3; 7.4)	3,821 (1.0)	2,029 (6.5)	5.2 (5.0; 5.5)^c^	6.7 (6.3; 7.1)
Any SUD	11,632 (3.2)	2,680 (14.4)	10.9 (10.4; 11.4)	4.5 (4.3; 4.7)^d^	15,617 (4.1)	4,954 (15.9)	11.4 (11.0; 11.8)	4.1 (3.9; 4.2)^d^

PD and HR estimates are shown (with 95% CI)

*Abbreviations*: *PD* Prevalence difference, *HR* Hazard ratio, *CI* confidence intervals, *SUD* Substance Use Disorder

^a^Adjusted for age (2-year categories)

^b^Adjusted for age (2-year categories) and mother’s education (low, middle, high)

^c^PDs tested for interaction between ADHD and sex on the additive scale were statistically significant (*p* < 0.05)

^d^HRs tested for interaction between ADHD and sex on the multiplicative scale were statistically significant (*p* < 0.05)

### Risk of SUD in individuals with versus without ADHD

Adjusted HR estimates (aHRs) are also shown in Table 2 (sex-specific) and Additional file 2 (total population). In contrast to when using absolute measures (PDs), ratio measures (aHR estimates) for alcohol-related disorders and “any SUD” were among the lowest across the various SUD diagnoses. The aHR estimates of other substances were higher, among females from aHR 6.5 (cannabis-related disorders) to aHR 8.0 (stimulant-related disorders) and among males from aHR 5.5 (cannabis-related disorders) to aHR 7.6 (opioid-related disorders). Sex-differences in aHR estimates were fewer and smaller than those of PDs, and associations were stronger in females than males: Compared to females and males without ADHD, females with ADHD had significantly higher aHR of cannabis-related disorders and “any SUD” than males with ADHD (cannabis: aHR_females_ 6.5; aHR_males_ 5.5; and “any SUD”: aHR_females_ 4.5; aHR_males_ 4.1).

### ADHD, comorbid psychiatric disorders, and association with SUD

**Fig. 2:**
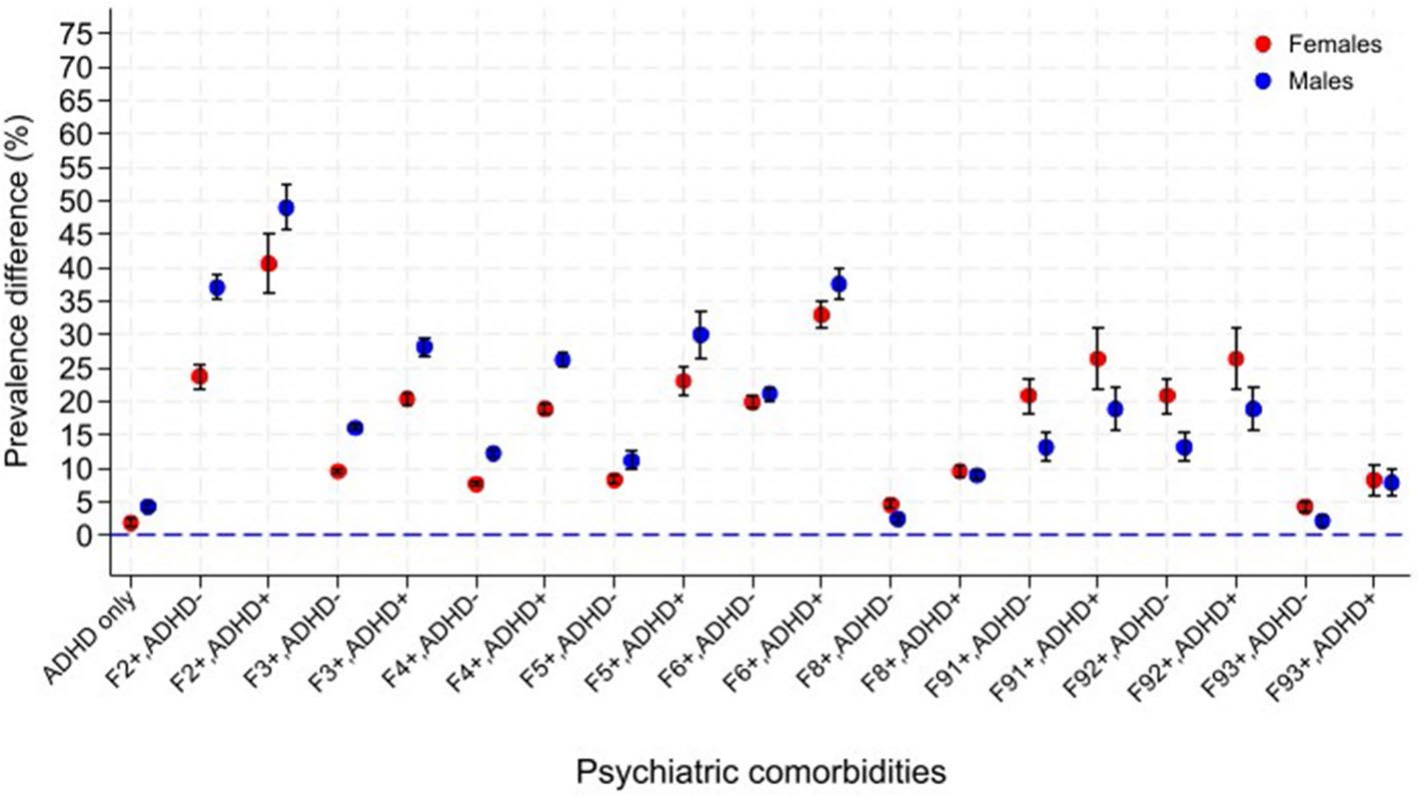
Prevalence differences for any SUD associated with ADHD and other psychiatric disorders. Legend: Age adjusted prevalence differences with 95% confidence intervals for any substance use disorder (any SUD) in males and females with ADHD only, ADHD with comorbid psychiatric disorders and psychiatric disorders without ADHD. Males and females with neither ADHD nor the other psychiatric disorders are reference groups. Shown for males (*N* = 412,714) and females (*N* = 387,473) born 1988–2001 and followed to 2019. Norway, 1988–2019

Figure 2 and Table 3 show sex-specific age-adjusted differences in prevalence of “any SUD” in individuals with ADHD, without and with specific comorbid psychiatric disorders, compared to individuals with neither ADHD nor any of the studied psychiatric disorders. In both males and females, the largest difference in prevalence was associated with the combination of ADHD and schizophrenia/psychosis (PD_females_: 40.6%; PD_males_: 49.0%). ADHD combined with personality disorders and eating/sleeping disorders were also associated with high PD estimates in both females and males (personality disorders: 33.0%_females_, 37.6%_males_; eating/sleeping disorders: 23.1%_females_, 30.0%_males_).

**Table 3:** Prevalence differences of SUD in males and females with and without ADHD and registered comorbid psychiatric disorders

Comorbidity	Females			Males		
	Total numbers	Any SUD; N (%)	PD (95% CI)^a^	Total numbers	Any SUD; N (%)	PD (95% CI)^a^
No ADHD, no other psych	382,009	13,997 (3.7)	0.0 (ref)	398,553	19,230 (4.8)	0.0 (ref)
ADHD, no other psych	5,464	315 (5.8)	1.9 (1.3; 2.5)	14,161	1,341 (9.5)	4.3 (3.9; 4.8)
ADHD + F2^b^	465	204 (43.9)	40.6 (36.1; 45.1)	825	439 (53.2)	49.0 (45.6; 52.4)
ADHD + F3^b^	6,663	1,510 (22.7)	20.4 (19.4; 21.4)	5,397	1711 (31.7)	28.2 (26.9; 29.4)
ADHD + F4^b^	8,585	1,801 (21.0)	18.9 (18.1; 19.8)	7,394	2,197 (29.7)	26.3 (25.2; 27.3)
ADHD + F5^b^	1,692	444 (26.2)	23.1 (21.0; 25.2)	718	247 (34.4)	30.0 (26.6; 33.5)
ADHD + F6^b^	2,263	814 (36.0)	33.0 (31.0; 35.0)	1615	678 (42.0)	37.6 (35.2; 40.0)
ADHD + F8^b^	4,158	529 (12.7)	9.6 (8.6; 10.5)	8,448	1093 (12.9)	9.0 (8.3; 9.7)
ADHD + F91^b^	476	133 (27.9)	24.9 (20.9; 28.9)	1180	323 (27.4)	23.5 (21.0; 26.0)
ADHD + F92^b^	389	115 (29.6)	26.4 (21.9; 30.9)	655	149 (22.8)	18.9 (15.8; 22.1)
ADHD + F93^b^	710	79 (11.1)	8.3 (6.0; 10.6)	939	108 (11.5)	7.9 (5.9; 9.8)
No ADHD, F2^b^	2,125	575 (27.1)	23.8 (21.9; 25.6)	2,923	1,211 (41.4)	37.1 (35.3; 38.9)
No ADHD, F3^b^	43,184	5,091 (11.8)	9.6 (9.3; 9.9)	21,459	4,233 (19.7)	16.1 (15.6; 16.7)
No ADHD, F4^b^	60,845	5,942 (9.8)	7.7 (7.5; 8.0)	29,856	4,724 (15.8)	12.3 (11.9; 12.7)
No ADHD, F5^b^	13,023	1,485 (11.4)	8.3 (7.8; 8.9)	2,514	394 (15.7)	11.2 (9.8; 12.6)
No ADHD, F6^b^	8,824	2,017 (22.9)	19.9 (19.0; 20.8)	5,407	1,388 (25.7)	21.2 (20.0; 22.3)
No ADHD, F8^b^	6,927	528 (7.6)	4.6 (4.0; 5.2)	10,949	710 (6.5)	2.5 (2.1; 2.9)
No ADHD, F91^b^	746	166 (22.3)	19.3 (16.3; 22.2)	1,508	296 (19.6)	15.9 (13.9; 17.9)
No ADHD, F92^b^	1,006	241 (24.0)	20.9 (18.3; 23.5)	1,182	201 (17.0)	13.2 (11.1; 15.3)
No ADHD, F93^b^	3,166	224 (7.1)	4.3 (3.4; 5.1)	2,413	133 (5.5)	2.2 (1.3; 3.0)

Prevalence differences (with 95% confidence intervals) for any diagnosis of substance use disorder (any SUD) in individuals with and without ADHD, without and with registered comorbid psychiatric disorders, compared to individuals with neither ADHD nor the other psychiatric disorders (reference group). Shown for males and females separately

*Abbreviations*: *PD* Prevalence difference, *CI* confidence intervals, *SUD* Substance Use Disorder

^a^Adjusted for age (2-year categories)

^b^ICD-10 codes for Psychiatric disorders: Schizophrenia/psychosis (F2), Mood/affective disorders (F3), Anxiety/somatoform disorders (F4), Eating/sleeping disorders (F5), Personality disorders (F6), Disorders of psychological development (F8), Conduct disorders (F91), Mixed disorders of conduct and emotion (F92), and Emotional disorders with childhood onset (F93)

When looking at sex-differences across the various combinations of ADHD and other psychiatric disorders, males had consistently larger PD estimates of “any SUD” than females when ADHD was combined with psychiatric disorders typically diagnosed in adulthood (schizophrenia/psychosis, mood/affective disorders, anxiety/somatoform disorders, eating/sleeping disorders, and personality disorders). On the other hand, when ADHD was combined with some of the disorders more typically diagnosed in childhood (conduct disorders and mixed disorders of conduct and emotion), the differences in prevalence were larger in females.

Among individuals without ADHD, the PD patterns of “any SUD” were very similar to those found for individuals with ADHD, but with lower PD estimates. The change in sex-differences across psychiatric disorders was also similar to the change in individuals with ADHD.

## Discussion

In this national registry-based study, we found that people with ADHD had higher rates of SUD than those without ADHD. These differences were larger in males than females for most types of SUD. The difference in prevalence of SUD was even higher when ADHD was combined with psychiatric disorders such as schizophrenia/psychosis or personality disorders. When ADHD was combined with psychiatric disorders usually diagnosed in adulthood, the differences in SUD prevalence were larger in males than females, while larger in females when ADHD was combined with some disorders typically diagnosed in childhood. The sex differences in relative measures (ratios) were smaller and fewer than in the absolute prevalence differences.

### Prevalence of SUD in individuals with versus without ADHD

Our finding that people with ADHD have higher rates of SUD than those without ADHD is consistent with previous studies [17, 22, 23, 34, 35, 39]. Impulsivity, a common trait in ADHD, might lead to experimenting with substances, explaining some of the association with SUD [49]. The misuse of stimulants in people with ADHD may represent an attempt of self-medication, although with conflicting evidence [50, 51], while other sedative drugs such as alcohol, cannabis, or opioids may also be used to counter periods of overactivation and relieve unpleasant feelings and symptoms, or to have euphoric experiences [52].

We found that the difference in rates of SUD in people with versus without ADHD were larger in males than females for most types of SUD. This matches previous studies using absolute rate differences of SUD in males and females with and without ADHD [17, 41]. The finding might partly be because females often get diagnosed with ADHD later than males [18, 53], thus SUD could be registered in females with undiagnosed ADHD and the association with ADHD would be missed. Additionally, females are generally less likely to receive treatment for SUD and not be registered with SUD as often as males [54].

We further found that males both with and without ADHD had higher rates of the different SUD subtypes than females. This aligns with reports on SUD rates among males and females in general. For example, in a World Drug Report from 2023, males are reported to inject drugs five times more often than females [54]. In our study, we found that opioid-related disorders were more common in males than females regardless of ADHD status, and there was no significant sex-difference in the association with ADHD.

### Risk of SUD in individuals with versus without ADHD

When we used relative effect measures (ratios), we found the weakest association between ADHD and alcohol-related disorders and SUD overall (any). This is probably because these disorders are more common in people without ADHD. When using relative effect measures (ratios), a higher rate in the reference group (the denominator) lowers the estimate. This probably also explains some of the differences between sexes when using relative estimates instead of absolute differences (PDs): Females had higher relative risks than males for cannabis-related disorders and SUD overall. Individuals with ADHD may experiment more with substances than those without ADHD, and this difference may be larger in females than males because females without ADHD may experiment with substances to a lesser degree [17, 34, 55]. Our findings here match a Danish study [34] reporting a higher relative risk of both cannabis and alcohol-related disorders in females with relative without ADHD compared to males before adjusting for other psychiatric conditions. The sex differences were, however, not significant after full adjustment. A later study by some of the same authors [40], reported higher relative risk of SUD overall in females than males with vs without ADHD, in agreement with Chen et al. [41]. The authors suggested this is because females with ADHD have less or later access to ADHD medication and higher rates of other psychiatric conditions, as also reported by others [17, 41, 53, 56].

### ADHD, comorbid psychiatric disorders, and association with SUD

When ADHD was combined with other psychiatric disorders, the differences in SUD rates in both males and females compared to males and females with neither ADHD nor the studied psychiatric disorders were even larger. Since we only know the timing of the first recorded SUD in specialist healthcare and we do not know the exact timing of when other psychiatric disorders started, the order of events is uncertain. Our data cannot verify to which degree ADHD increases the risk of another psychiatric disorder that could then lead to SUD, or whether ADHD directly increases the risk of SUD, which then leads to another psychiatric disorder. However, when compared to people with neither ADHD nor the other psychiatric disorders, we found that the differences in SUD prevalence were larger in people with both ADHD and other psychiatric disorders than in those with the same psychiatric disorders but without ADHD. This suggests that males and females with ADHD need close monitoring for both SUD and other psychiatric disorders.

In our study, the difference in prevalence of SUD overall was particularly high in individuals with ADHD and schizophrenia/psychosis, especially in males. Schizophrenia/psychosis alone was also associated with significantly higher rates of SUD, particularly in males, but clearly lower than when ADHD was also present. SUD is often linked to schizophrenia/psychosis because SUD can cause psychosis, but also because people with schizophrenia/psychosis are at higher risk for SUD [17, 57, 58]. Our findings suggest that both males and females with ADHD should be closely monitored for the combination of schizophrenia/psychosis and SUD.

Further, individuals with ADHD, especially males, had larger differences in rates of SUD when they also had personality disorders or eating/sleeping disorders. Other studies have found strong links between SUD and personality disorders [59], eating disorders [60], and sleeping disorders [61]. ADHD increases the risk of these psychiatric disorders [17, 62, 63], which may explain why we see higher rates of SUD in individuals with ADHD and these psychiatric comorbidities compared to those with neither ADHD nor the other psychiatric disorders.

An interesting and novel finding in our study was the sex differences in associations between ADHD and SUD across the specific comorbid psychiatric disorders: Compared to the reference group, males had larger differences in SUD rates than females when ADHD was combined with psychiatric disorders typically diagnosed in adulthood, while females had larger rate differences when ADHD was combined with some childhood disorders, e.g. conduct disorders. This pattern was also seen in individuals with psychiatric disorders without ADHD. Boys with disruptive behavior are more likely to be diagnosed with ADHD, while girls with similar behavior may receive other behavioral diagnoses, like conduct disorders as a child [64] and ADHD at an older age. Further, females with disruptive behavior in childhood are reported to have a high risk of SUD [55]. For adult disorders, the higher differences in SUD rates in males align with the general trend of higher SUD prevalence in males, as also shown in our study.

### Strengths and limitations

Our study has several strengths. It includes a large population-based sample of children, adolescents and young adults clinically diagnosed with ADHD, with or without SUD and other psychiatric comorbid disorders. Our cohort includes individuals covered by information about SUD and other psychiatric disorders from the age of 7 to 31 years, which is a relevant age span to capture both childhood psychiatric disorders and the first treatment of SUD in specialist health care. Due to the inclusion of a large proportion of females, we could evaluate sex-differences in less prevalent psychiatric disorders, such as specific SUD types and ADHD comorbid with other psychiatric disorders. Unlike other large population studies, we did not use symptom ratings or self-reports to define the diagnosis. The data are collected from nationwide health registries of good quality and with mandatory reporting [44–46, 48]. ADHD was defined using data from registries covering dispensed ADHD medication and/or an ADHD diagnosis from specialist health care. In Norway, medication for ADHD is only initiated after careful diagnostic evaluation by a specialist in psychiatry, addictions, or child- and adolescent psychiatry [65].

However, several limitations must also be mentioned. Although the health registries are shown to have good quality in many studies, the validity of diagnoses in the NPR may vary depending on the specific diagnosis [46], also reported with regards to severe mental disorders [66]. We lack insight into diagnostic practices behind the registry codes, which further may have changed from 2008 to 2019. The clinical challenge of finding a correct diagnosis for psychiatric symptoms will also affect the reliability of diagnoses in the NPR. Further, the comorbidity reported may not represent simultaneous disorder onset, as we report comorbidities registered at some point during 2008–2019. However, our youngest individuals were 7 years in 2008 and 18 in 2019 while the oldest were 20 years in 2008 and 31 in 2019. We therefore assume that comorbidities registered in the specific individual will likely represent disorders diagnosed close in time.

Another limitation is that ADHD was defined based on dispensed ADHD medication available from 2004 (NorPD) and diagnosis available from 2008 (NPR). A large proportion of our ADHD individuals are defined by dispensed ADHD medication alone. One potential contradiction for using ADHD medication is active SUD [67] and it is therefore a limitation that we do not have information on both medication and diagnosis covering the same years. Further, with information about ADHD from 2004 but SUD and the psychiatric disorders from 2008, we are not able to identify with certainty which diagnosis came first. Since ADHD is viewed as a highly heritable neurodevelopmental disorder with childhood onset [8, 10], we may assume that ADHD symptoms and in most cases also the diagnosis—were present before the SUD diagnosis. However, further investigation is needed to evaluate temporal associations between the diagnoses, especially with regards to sex-differences, since many females get their ADHD diagnosis at an older age than males [18, 19, 53]. How ADHD symptoms without a diagnosis and thus without getting treatment may influence the risk of SUD is an important aspect for further study, this is important also in relation to the reported sex-differences in PDs.

The higher SUD prevalence among individuals with than without ADHD may partly be explained by a diagnostic bias since individuals with ADHD are already in contact with the health service. This is also relevant for the other psychiatric disorders covered. The differences in prevalence were in line with results presented in several previous studies [17, 22, 23, 33–35, 39], but this bias may still have impacted our results leading to overestimated prevalence differences. Further, some individuals with SUD in the non-ADHD group could have undetected ADHD, especially females who tend to get their ADHD diagnosis later than males. This could lead to an underestimation of PDs, especially in females. Disorders treated only in primary health care are not covered in our data, however most of the studied disorders are likely linked to tertiary/specialist care at some stage. Less severe disorders and SUD especially in the youngest part of our cohort (before being referred to specialist care) will, however, more often be missed.

Finally, we studied the psychiatric group eating/sleeping disorders (F5) as one entity, while in fact, these are quite different disorders. This was done to include a large enough sample size to make meaningful analyses. There were for example only two males with ADHD, eating disorders and “any SUD”.

### Clinical implications

The overall aim of the current study was to identify groups that may profit from closer follow-up and interventions to prevent and treat SUD. In general, both males and females with ADHD had higher SUD prevalence especially when comorbid with other psychiatric disorders compared to their reference groups. Although the difference in prevalence of SUD in individuals with than without ADHD was higher in males than females, the sex-difference was not large, making prevention relevant for both sexes. The results also point to younger individuals with ADHD as an important target group for close follow-up with regards to both prevention of SUD and earlier interventions for other psychiatric comorbid disorders. For girls diagnosed with conduct disorders and mixed disorders of conduct and emotion, follow-up should be with regards to both SUD and a possibly underlying ADHD. Collaboration between health services and schools is relevant here, although studies show varying results regarding school engagement in prevention of SUD [68, 69].

## Conclusion

In this registry-based study covering the total Norwegian population aged 7 to 31 years, ADHD was strongly associated with SUD across all substances among both males and females with stronger associations in males than females for most specific SUD-groups. ADHD with comorbid psychiatric disorders, especially schizophrenia and personality disorders, were associated with the largest differences in SUD rates when compared to individuals without ADHD and the studied psychiatric disorders. Sex-differences in associations between ADHD and SUD when ADHD was comorbid with other psychiatric disorders were dependent on the specific psychiatric disorder. Further studies are needed to evaluate how the temporal associations between ADHD and SUD may differ between females and males, including the impact of delayed ADHD diagnosis which is more common in females. However, the results point to younger males and females with ADHD as important target groups for close follow-up to prevent development of later comorbid disorders, including SUD and severe psychiatric disorders. This is relevant for all clinicians diagnosing and treating ADHD.

## Supplementary Information

Additional file 1. Age adjusted prevalence rates of specific SUD groups in males and females with and without ADHD.

Additional file 2. Prevalence differences and hazard ratios of SUD in individuals with and without ADHD.

## Data Availability

The datasets generated and analysed during the current study are not publicly available due to ethical and legal restrictions in Norway: they are defined as being potentially identifying and they include sensitive information. Restrictions apply to the availability of such data according to the license from the Regional Committee on Medical and Health Research Ethics and the Health Research Act (ACT 2008-06-20 no. 44: Act on medical and health research). Data are, however, available for other researchers upon request to the Norwegian Institute of Public Health (https://helsedata.no/) under the approval of the Regional Ethic Committee (ethical consent: https://rekportalen.no/#hjem/home).

## Abbreviations

SUD: Substance use disorder
ADHD: Attention deficit/hyperactivity disorder
MBRN: The Medical Birth Registry of Norway
NorPD: The Norwegian Prescription Database
NPR: The Norwegian Patient registry
ATC: Anatomical Therapeutic Chemical
ICD: International classification of diseases
PD: Prevalence difference
HR: Hazard ratio
CI: Confidence interval
*p-value*: Probability value
(F10) Alcohol: Mental and behavioral disorders due to use of alcohol
(F11) Opioids: Mental and behavioral disorders due to use of opioids
(F12) Cannabis: Mental and behavioral disorders due to use of cannabinoids
(F13) Sedatives: Mental and behavioral disorders due to use of sedatives or hypnotics
(F14) Cocaine: Mental and behavioral disorders due to use of cocaine
(F15) Other stimulants: Mental and behavioral disorders due to use of other stimulants, including amphetamines and caffeine
(F16) Hallucinogens: Mental and behavioral disorders due to use of hallucinogens
(F18) Inhalants: Mental and behavioral disorders due to use of volatile solvents
(F19) Multiple psychoactive substance-related disorders: Mental and behavioral disorders due to multiple drug use and use of other psychoactive substances
(F2) Schizophrenia: Schizophrenia, schizotypal and delusional disorders
(F3) Mood/affective disorders: Mood [affective] disorders
(F4) Anxiety/somatoform: Neurotic, stress-related and somatoform disorders
(F5) Eating/sleeping disorders: Behavioral syndromes associated with physiological disturbances and physical factors
(F6): Personality disorders
(F8): Disorders of psychological development, including learning disabilities and autism spectrum disorders
(F91): Conduct disorders
(F92): Mixed disorders of conduct and emotion
(F93): Emotional disorder with onset specific to childhood
