# Supplementary material for: Prevalence of substance use disorder in individuals with attention deficit/hyperactivity disorder: associations with sex and psychiatric comorbidity
Source: BMC Psychiatry. 2025 Oct 7;25:936. doi: 10.1186/s12888-025-07305-1 (PMC12505809; doi:10.1186/s12888-025-07305-1)
Supplement: Supplementary file 1 — Additional file 1. Age adjusted prevalence rates of specific SUD groups in males and females with and without ADHD. [file 12888_2025_7305_MOESM1_ESM.pdf]

Additional table 1: Age adjusted prevalence rates of specific SUD groups in males and females with and without ADHD.

Substance	Age adjusted [a] prevalence rates (%) with 95% confidence intervals			
	Non-ADHD		ADHD	
	Females	Males	Females	Males
Alcohol-related disorders (F10)	2.15 (2.11; 2.20)	7.91 (7.65; 8.18)	2.42 (2.37; 2.47)	8.89 (8.62; 9.15)
Opioid- related disorders (F11)	0.21 (0.20; 0.22)	1.68 (1.54; 1.81)	0.27 (0.25; 0.28)	2.13 (1.98; 2.28)
Cannabis- related disorders (F12)	0.77 (0.75; 0.80)	4.57 (4.37; 4.76)	1.62 (1.58; 1.66)	9.58 (9.27; 9.88)
Sedatives- related disorders (e.g., benzodiazepines) (F13)	0.42 (0.40; 0.43)	3.15 (2.97; 3.33)	0.52 (0.50; 0.54)	3.92 (3.72; 4.11)
Stimulant- related disorders (e.g., cocaine and amphetamines) (F14-F15)	0.47 (0.45; 0.49)	3.69 (3.49; 3.88)	0.63 (0.61; 0.66)	5.02 (4.80; 5.24)
Other substance- related disorders (F16, F18)	0.06 (0.05; 0.06)	0.37 (0.31; 0.43)	0.12 (0.11; 0.13)	0.78 (0.69; 0.87)
Multiple psychoactive substance-related disorders (F19)	0.74 (0.72; 0.77)	5.26 (5.03; 5.49)	0.99 (0.95; 1.02)	7.02 (6.77; 7.28)
Any SUD	3.20 (3.15; 3.25)	13.47 (13.14; 13.81)	4.04 (3.98; 4.10)	17.01 (16.65; 17.37)

Abbreviations: CI: confidence intervals, SUD: Substance Use Disorder

[a] Adjusted for age (2-year categories).
