# Supplementary material for: Prevalence of substance use disorder in individuals with attention deficit/hyperactivity disorder: associations with sex and psychiatric comorbidity
Source: BMC Psychiatry. 2025 Oct 7;25:936. doi: 10.1186/s12888-025-07305-1 (PMC12505809; doi:10.1186/s12888-025-07305-1)
Supplement: Supplementary file 2 — Additional file 2. Prevalence differences and hazard ratios of SUD in individuals with and without ADHD. [file 12888_2025_7305_MOESM2_ESM.pdf]

Additional table 2: Prevalence differences and hazard ratios of SUD in individuals with and without ADHD.

Substance	Non-ADHD N (%)	ADHD N (%)	PD[a] (95% CI)	HR[b] (95% CI)
Alcohol-related disorders (F10)	17,196 (2.3)	4,180 (8.4)	5.9 (5.6; 6.1)	3.6 (3.4; 3.7)
Opioid-related disorders (F11)	1,789 (0.2)	925 (1.9)	1.5 (1.4; 1.6)	7.5 (6.9; 8.1)
Cannabis-related disorders (F12)	9,061 (1.2)	3,711 (7.5)	5.9 (5.7; 6.1)	5.7 (5.5; 5.9)
Sedative-related disorders (e.g., benzodiazepines) (F13)	3,512 (0.5)	1,731 (3.5)	2.8 (2.7; 3.0)	7.2 (6.7; 7.6)
Stimulant-related disorders (e.g., cocaine and amphetamines) (F14-F15)	4,153 (0.6)	2,155 (4.3)	3.6 (3.4; 3.8)	7.5 (7.1; 7.9)
Other substance- related disorders (F16- F18)	675 (0.1)	304 (0.6)	0.5 (0.4; 0.6)	6.0 (5.2; 6.9)
Multiple psychoactive substance-related disorders (F19)	6,523 (0.9)	3,048 (6.1)	5.0 (4.8; 5.2)	6.7 (6.4; 7.0)
Any SUD[d]	27,249 (3.6)	7,634 (15.3)	11.3 (10.9; 11.6)	4.2 (4.1; 4.3)

Abbreviations: PD: Prevalence difference, HR: Hazard ratio, CI: confidence intervals, SUD: substance use disorder
Notes: PD and HR estimates are shown (with 95% CI)

[a] Adjusted for age (2-year categories).

[b] Adjusted for age (2-year categories), sex and mother's education (low, middle, high)
